# Bilateral Cross-Modal Fusion Network for Robot Grasp Detection

**DOI:** 10.3390/s23063340

**Published:** 2023-03-22

**Authors:** Qiang Zhang, Xueying Sun

**Affiliations:** 1 School of Automation, Jiangsu University of Science and Technology, No. 666 Changhui Road, Zhenjiang 212100, China; 2 Systems Science Laboratory, Jiangsu University of Science and Technology, No. 666 Changhui Road, Zhenjiang 212100, China

**Keywords:** robot grasp detection, cross-modality fusion, channel interaction

## Abstract

In the field of vision-based robot grasping, effectively leveraging RGB and depth information to accurately determine the position and pose of a target is a critical issue. To address this challenge, we proposed a tri-stream cross-modal fusion architecture for 2-DoF visual grasp detection. This architecture facilitates the interaction of RGB and depth bilateral information and was designed to efficiently aggregate multiscale information. Our novel modal interaction module (MIM) with a spatial-wise cross-attention algorithm adaptively captures cross-modal feature information. Meanwhile, the channel interaction modules (CIM) further enhance the aggregation of different modal streams. In addition, we efficiently aggregated global multiscale information through a hierarchical structure with skipping connections. To evaluate the performance of our proposed method, we conducted validation experiments on standard public datasets and real robot grasping experiments. We achieved image-wise detection accuracy of 99.4% and 96.7% on Cornell and Jacquard datasets, respectively. The object-wise detection accuracy reached 97.8% and 94.6% on the same datasets. Furthermore, physical experiments using the 6-DoF Elite robot demonstrated a success rate of 94.5%. These experiments highlight the superior accuracy of our proposed method.

## 1. Introduction

In the realm of robotics, the advancement of intelligence has significantly boosted the adoption of robots. As a result, the visual detection of targets has become an increasingly crucial area of focus in robotics research. A robot’s ability to grasp and transport objects, either independently or in response to user commands, can enhance its ability to assimilate into the environment and broaden the range of potential robotic applications. Presently, the utilization of RGB-D cameras is making remarkable strides in robot grasping, thanks to the evolution of vision sensor technology.

Our work focuses on RGB-D data-driven robot grasp detection. Many pioneers in the field have achieved remarkable results. In the past decade, convolutional neural networks (CNNs) [1,2,3,4,5] have become the most widely utilized solution for robot grasp detection due to their superiority in feature representation, resulting in outstanding detection accuracy and high efficiency. While CNNs excel at local feature representation, they tend to lose information with global relevance. Recently, transformer-based approaches have gained significant popularity for visual tasks and have demonstrated comparable or superior performance in classification, semantic segmentation, and object detection. Some researchers, such as S. Wang et al. [6], have demonstrated the applicability of transformers in robot grasp detection.

Despite the impressive strides made by deep learning in solving the problem of visually detecting and grasping targets, the robustness of grasp detection still requires further improvement. This is because, while either RGB or depth images can provide some information about the scene, they are only partial in nature and may not always be effective in obtaining reliable detection results across different scenarios. Therefore, it is essential to leverage the information provided by both modalities to enhance grasp detection.

To address this issue, researchers in the field have developed early [3,7,8,9] and late [2,10] multimodal fusion approaches for grasp detection. While these methods have yielded meaningful results, the correlation between multimodal data has only been partially exploited. Recent studies have focused on exploring the mechanisms of intermediate fusion [11,12]. Although these methods have improved the efficiency of RGB and depth modalities in robot grasp detection, making the most of the bilateral modal information still remains a challenge.

To solve the problem of multimodal fusion, we proposed a tri-stream cross-modal fusion architecture to achieve bilateral information interaction. The key idea was to use the proposed MIM approach to capture the global association information between modalities. Subsequently, the aggregation of different modal streams was refined through adaptive CIM units. The main contributions of our work can be summarized as follows:

- We proposed a tri-stream cross-modal fusion architecture to facilitate the interaction of RGB and depth bilateral information and efficiently aggregate multiscale information;
- A novel spatial-wise cross-attention algorithm was developed to adaptively capture cross-modal feature information. The channel interaction modules further enhanced the aggregation of different modal streams;
- The proposed method demonstrated state-of-the-art grasp detection accuracy on both the Cornell and Jacquard datasets, with image-wise detection accuracy reaching 99.4% and 96.7% on Cornell and Jacquard, respectively, with object-wise detection accuracy reaching 97.8% and 94.6% on the same datasets;
- The proposed method has also shown success in guiding gripping tasks in the real world, achieving a 94.5% success rate on household items.

The remaining parts of the article are structured as follows. Section 2 presents the deep regression model for detecting robot grasps. Section 3 describes the formulation of grasp detection. The proposed method is elaborated in detail in Section 4. The performance evaluation of the proposed method is presented in Section 5. Finally, Section 6 provides a summary and conclusion of the article.

## 2. Related Works

### 2.1. Grasp Model Representation

The representation of the robot grasp model is a prerequisite for identifying the gripping position. In vision-based approaches, the object grasp can be divided into the 2-DoF planar grasp and the 6-DoF grasp, based on various application scenarios. For instance, 2-DoF planar grasp implies that the target object is positioned on a flat working surface and is confined from one direction. Thus, the grasping information is reduced from 6D to 3D, specifically 2D in-plane position and 1D rotation angle. On the other hand, 6-DoF grasping enables the gripper to hold objects from different angles in the 3D space.

In 2006, A. Saxena et al. [13] proposed a point-based model representation for 6-DoF grasp detection. This representation considers the target location to be a point in 3D space. The point is detected in the image, and the relative position of the point with respect to the robot end effector is estimated using either a binocular camera or motion recovery structure, enabling the robot to perform the grasp operation. In 2010, Q.V. Le et al. [14] proposed a multi-points linkage approach to express the grasp position. Subsequent studies, such as those outlined in references [14,15,16,17], have achieved significant progress in terms of detection accuracy, reliability, and efficiency.

For 2-DoF planar grasp detection, Y. Jiang [18] proposed a rectangular representation method for the robot grasp that bypasses the object detection and pose estimation process. In this method, each grasp is represented by a rectangle with its central coordinates, width, height, and rotation angle. This simplifies the model’s complexity significantly. Since then, many researchers, such as [2,3,4,5,9,14,19,20], have focused on robot grasp detection using the rectangular model, and these studies have made efforts to improve the robustness of feature representation and the real-time performance of detection.

### 2.2. 2-DoF Planar Grasp Detection Approaches Based on Rectangular Representation

In recent years, convolutional neural networks [21] have become the most widely utilized solution for vision tasks. When it comes to studies on robot grasp detection, many researchers have focused on improving the quality of deep neural networks [22,23] in order to achieve better detection results. One such method was proposed by I. Lenz et al. [1] in 2014, which utilized a multilayer deep self-encoder for image feature extraction in combination with a support vector machine classifier. J. Wei et al. [24] proposed a similar approach in 2017 using the Deep Extreme Learning Machine for automatic encoding. Trottier et al. [25] also proposed a detection method in the same year using a self-coding dictionary learning method with a support vector machine classifier, although it was found to be slow and not well-suited for robotic object grasping. Z. Wang et al. [26] proposed a unified model for object segmentation and grasp detection in 2016. The method combined a grasping detection network with a two-stage estimator to improve detection accuracy.

Due to the complexity of multi-stage detection methods, more researchers are focusing on the end-to-end approach. In 2015, J. Redmon et al. [27] proposed a robot grasp detection method based on multilayer convolutional neural networks, which allowed for end-to-end training and reduced manual involvement in the training process. This approach also significantly improved detection efficiency through direct regression.

In 2018, heatmap regression methods were first utilized by D. Morrison et al. [4] to indirectly obtain grasp detection results. In their follow-up study, D. Morrison et al. [5] introduced a generative convolutional neural network for robot grasp detection. In 2020, S. Kumra et al. [8] proposed an antipodal robotic grasp detection method using a residual convolutional neural network, achieving image-wise detection accuracy of 97.7% and 94.6% on Cornell and Jacquard datasets. Their work was further improved upon in 2022 by the same group [20]. H. Cao et al. [3] proposed an efficient convolutional neural network using Gaussian-based grasp representation in 2021, which achieved image-wise detection accuracy of 97.8% and 95.6% for Cornell and Jacquard datasets, respectively. Lastly, S. Ainetter and F. Fraundorfer [28] proposed an end-to-end method for robot grasp detection in 2021, using a semantic segmentation refinement engine to increase detection accuracy.

A recent development in the field of robot grasp detection is the transformer-based method proposed by S. Wang et al. [6]. In their study, they made a preliminary attempt to address the 2-DoF grasp detection problem using the transformer architecture, and achieved impressive detection accuracy and efficiency, proving to be a competitive method in the field.

Our work explored the effectiveness of hybrid models that integrate convolutional neural networks and transformer architectures to detect 2-DoF robot grasping. This approach offers new insights into the design of effective grasp detection systems.

### 2.3. Multiple Modality Fusion Based Grasp Detection

With the wide adoption of RGB-D sensors, an increasing number of studies have turned their attention to the efficient fusion of multimodal data. Various approaches for multimodality fusion have been proposed, including early-fusion, late-fusion, and intermediate-fusion techniques.

In 2018, F. Chu et al. [7] introduced an early-fusion approach that integrated R, G, and depth channels to predict multi-grasps for multiple objects. Two years later, in 2020, S. Kumra et al. [8] presented a generative residual convolutional neural network for grasp detection, utilizing an early-fusion strategy with both RGB and depth images. Similarly, in 2022, H. Cao et al. [3] proposed a Gaussian-based grasp representation method using a generative grasping detection model that incorporated both RGB and depth images as inputs. Also in 2022, S. Yu et al. [9] introduced another approach using a residual neural network and squeeze-and-excitation modules.

In 2017, Q. Zhang et al. [2] put forth a sturdy robot grasp detection method that integrated RGB and depth features in the prediction head based on the YOLO architecture [29]. In 2022, Y. Song et al. [10] also proposed a hierarchical late-fusion method for RGB-D data, utilizing two CNN branches in the form of U-Net [30]. The decoding process hierarchically merged the RGB and depth features.

In 2022, K. Song et al. [31] proposed a triple-modal fusion architecture for robotic visual perception applications. In their work, a hierarchical weighted suppress interference approach was introduced to achieve robust features. H. Tian et al. [11] introduced an intermediate-fusion method for lightweight pixel-wise robot grasp detection, utilizing RGB and depth information. In 2023, H. Tian et al. [12] extended their work by introducing a rotation adaptive grasp detection approach, which also utilizes intermediate data fusion. They achieved a remarkable state-of-the-art accuracy of 99.3% and 94.6% on the Cornell and Jacquard datasets, respectively.

Research on multimodal fusion has yielded promising results. However, the effectiveness of cross-modal fusion is still limited by the quality of bilateral mutual information support. To address this issue, we propose a novel solution in this paper. Our approach offers an improved framework for cross-modal fusion that enhances the mutual information support between modalities and enables more effective integration of multimodal data. Our results demonstrate the viability and superiority of our proposed method in achieving better performance in robot grasp detection.

## 3. Problem Formulation

The robot is capable of using different types of grippers, including two-finger, three-finger, or multi-finger grippers, to grasp objects. However, parallel two-finger grippers are commonly preferred due to their simple design and cost-effectiveness. For 2-DoF grasp applications, a grasp can be represented by a 5-dimensional tuple *g* = {*x, y, θ, w, h*} [1,8,27]. The tuple g describes a rectangle with the center coordinates (*x*, *y*), the gripper height size (*h*), the gripper opening distance (*w*), and the orientation of the grasp rectangle (*θ*) with respect to the horizontal axis. Typically, the gripper dimensions are known, which allows the grasp representation to be simplified to *g* = (*x, y, θ, w*).

Instead of the 5-dimensional representation, D. Morrison et al. [5] provide an improved version of a grasp described as follows:

(1) $$\tilde{G}=\left(\tilde{Q},\tilde{\emptyset },\tilde{W}\right)\in {\mathbb{R}}^{3\times H\times W}$$

In Equation (1), ${\tilde{Q}}_{i,j}\in \left[0,\;1\right]$ denotes the detection quality of each pixel in the image, while ${\tilde{\emptyset }}_{i,j}\in \left[-\pi /2,\;\pi /2\right]$ represents the rotation angle of the gripper, and ${\tilde{W}}_{i,j}\in \left[0,W_{\max}\right]$ specifies the required width of the gripper’s opening. Our work involves the transformation of the grasp detection problem into a pixel-level prediction. Specifically, we propose a cross-modal fusion method to derive $\tilde{G}$ from an RGB-D image of the environment in which the grasping targets are located.

Equation (1) provides a comprehensive representation of the grasp image, but the rotation angle of the grip is challenging to determine due to its symmetrical values. To address this ambiguity, we encode the rotation angle using $\sin\left(2{\tilde{\emptyset }}_{i,j}\right)$ and $\cos(2{\tilde{\emptyset }}_{i,j}$), which helps eliminate any discontinuities that may arise during the calculation. The angle of the grasp to be executed at each pixel can be obtained using Equation (2):

(2) $${\tilde{\emptyset }}_{i,j}=\frac{1}{2}\times \arctan\left[\frac{\sin\left(2{\tilde{\emptyset }}_{i,j}\right)}{\cos\left(2{\tilde{\emptyset }}_{i,j}\right)}\right]$$

The optimal grasp within an image space is determined by identifying the pixel with the highest quality score in $\tilde{G}$. Additionally, the grasp can be straightforwardly mapped to physical space based on the internal and external parameters of the RGB-D camera.

## 4. Approach

### 4.1. Overview of Bilateral Cross-Modal Fusion Network

**Figure 1:**
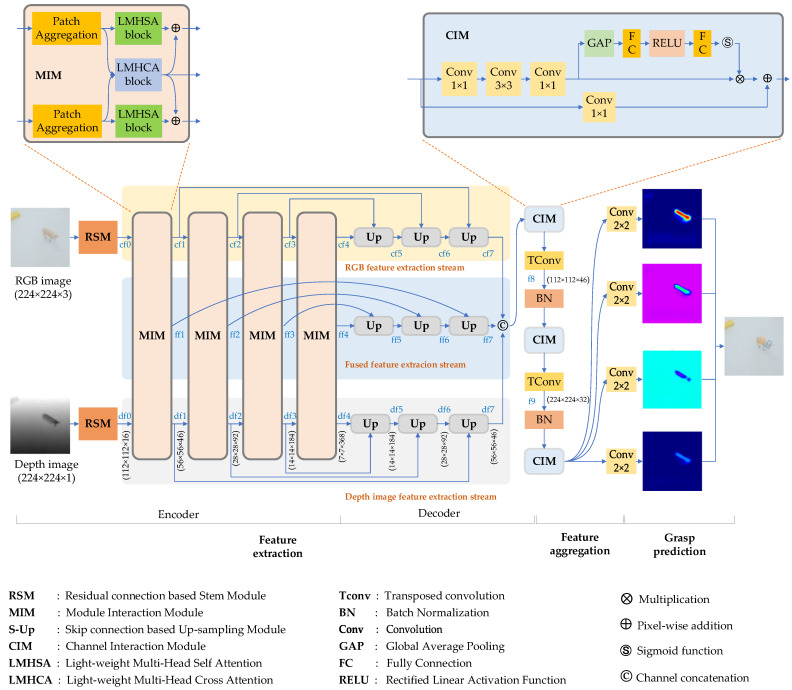
Bilateral cross-modal fusion network architecture.

Our robot grasp detection architecture is illustrated in Figure 1. It comprises three main components: feature extraction, feature aggregation, and grasp prediction. To ensure robust feature extraction, we employed two strategies. First, we tackled the problem of modality interaction in feature fusion by assigning adaptive weights to RGB and depth image features during the fused feature extraction stage. Second, we adopted a channel interaction approach for feature aggregation.

The feature extraction method serves two purposes: extracting multi-scale features from the RGB and depth scene images and constructing fused features from the two modalities. This process is accomplished through three streams: the RGB feature extraction stream, the depth feature extraction stream, and the fused feature extraction stream. Within each stream, the feature extraction process comprises two key stages: feature encoding and decoding.

The architecture of the RGB and depth feature extraction process, as shown in Figure 1, is based on U-Net-like structures. Similar to the approach in [32], each stream consists of a residual connection based stem module (RSM) and a series of modal interaction modules (MIM) for encoding RGB and depth features. Down-sampling operations are used to obtain multi-scale features. However, low-level features may contain more details but also unnecessary information, while high-level features may have more semantic information but may not represent small target features well. To obtain more robust features, we employ feature decoders, which are composed of transposed convolution and up-sampling processes with skip connections.

As depicted in Figure 1, the initial step in the feature extraction process involves the utilization of the RSM on the RGB image, which is responsible for extracting fine-grained features and generating the cf0 feature. To produce features at multiple scales, the cf0 feature undergoes several stages of light-weight multi-head self-attention modules (LMHSA) to create multi-scale features, including cf1, cf2, cf3, and cf4. These encoding procedures also involve the fused features from using an addition operation. To ensure that the resulting feature map is consistent with the size of the input image, a series of up-sampling modules are employed at the decoding step. Additionally, concatenation operations are incorporated into these processes to fully exploit the low-level and high-level features, leading to the production of the cf5, cf6, and cf7 feature maps. The depth feature extraction stream is also capable of generating corresponding feature maps, ranging from df0 to df7.

Compared to the encoding processes for RGB and depth features, the fused feature maps (ff1, ff2, ff3, and ff4) are initially generated using light weight multi-head cross-attention (LMHCA) strategy. During feature fusion, adaptive weights are assigned to the RGB and depth information. Details about the LMHCA algorithm is described in Section 4.2.2. To ensure the resulting fused features are robust, the same decoders used in the RGB and depth feature decoding processes are employed. Consequently, ff7 is of the same size as cf7 and df7.

As previously stated, the feature extraction process results in three distinct feature maps: cf7, df7, and ff7. It is our hypothesis that the feature aggregation process should optimally utilize useful information from all three features while minimizing the impact of irrelevant information. Previous research, such as that conducted in [2,11], has made numerous attempts to explore this topic. However, both studies employ equal-weight feature aggregation for each channel. In an effort to enhance the efficacy of feature aggregation, we have implemented a channel interaction strategy. As illustrated in Figure 1, the RGB, depth, and fused feature maps are initially concatenated. CIM units are then utilized to assign adaptive weights to the feature channels. Subsequent transposed convolution based up-sampling processes further improve the resolution of the fused feature map.

The grasp prediction head is comprised of several convolution calculation modules and is able to predict grasp quality, $\cos\left(2{\tilde{\emptyset }}_{i,j}\right)$, $\sin\left(2{\tilde{\emptyset }}_{i,j}\right)$, and grasp opening width heatmaps, which are employed to construct grasp rectangles.

### 4.2. Feature Extraction Pipeline

#### 4.2.1. Residual Connection Based Stem Module (RSM)

In our previous study, we discovered that feeding raw data directly into the transformer layer and training the neural network with the stochastic gradient descent (SGD) optimizer results in a high dependency on initialization seeds, resulting in challenging training and slow convergence. Drawing upon the insights provided by articles [32,33], we devised the RSM module, which renders the training process less sensitive to the pre-set hyperparameters and fosters network convergence. Moreover, RSM can effectively reduce data dimensionality with minimal computational overhead.

The residual network has proven to be highly effective in various applications such as image classification, object detection, and moving object tracking. To capitalize on its exceptional performance, we adopt a RSM module to generate a compact feature representation, thereby addressing the inferior feature representation capability of the linear projection.

**Figure 2:**
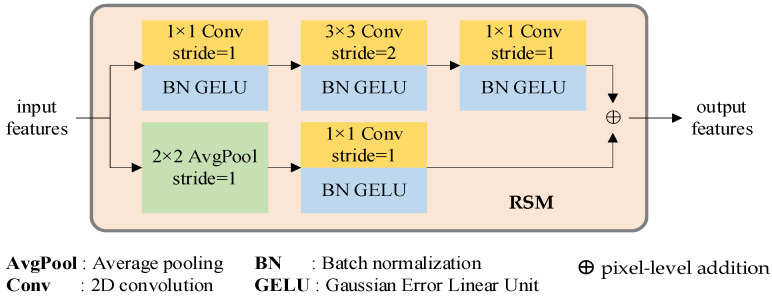
Input data feature extraction diagram using RSM.

Taking inspiration from [34], the stem module comprises two streams, as depicted in Figure 2. The first stream is composed of a sequence of convolution, Gaussian error linear unit (GELU) [35], and batch normalization (BN) [36] processes. The convolution modules utilize 1 × 1 (stride = 1), 3 × 3 (stride = 2), and 1 × 1 (stride = 1) kernels, respectively. This approach ensures that no information is neglected. The second stream includes a 2 × 2 (stride = 1) average pooling stage, a 1 × 1 (stride = 1) kernel convolution, GELU, and BN modules. This mechanism enables the expression of features while incurring minimal computational costs. The outputs of these two streams are combined to generate the encoding features, which are then fed into the self-attention and cross-attention procedures.

#### 4.2.2. Cross-Modal Feature Encoding Based on MIM

**Figure 3:**
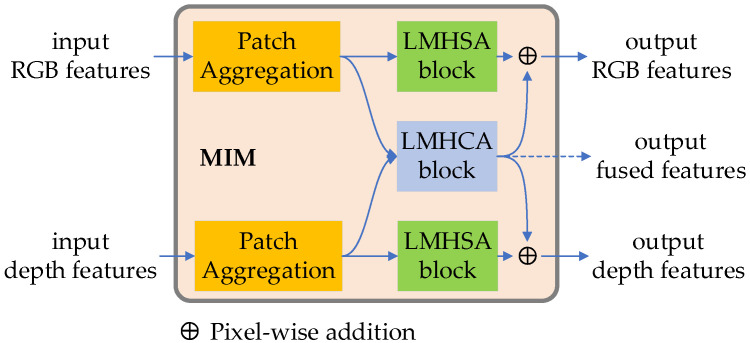
The structure of the MIM module.

The proposed MIM module is used to execute feature extraction and bilateral RGB and depth cross-modal fusion strategies. As shown in Figure 3, Each MIM module consists of two patch embedding blocks, two LMHSA blocks, one LMHCA module and two summation units. The patch aggregation module, composed by a 2 × 2 convolution with the stride = 2 and layer normalization block, is used to aggregate patches into a single image and produce hierarchical representation. The LMHSA module is used to extract features by spatial-wise self-attention. The LMHCA module is to compute fused features based on cross-attention. The summation operation helps to achieve mutual information support.

- Light weight multi-head self-attention (LMHSA) block

**Figure 4:**
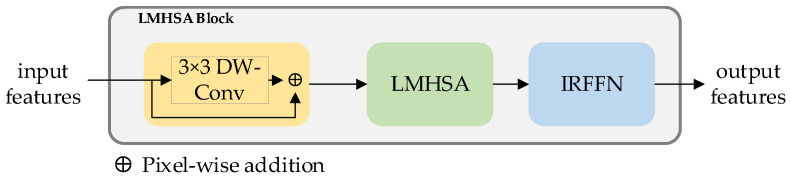
LMHSA block for RGB and Depth features extraction.

As shown in Figure 4, the LMHSA block contains three parts, which are local perception, LMHSA and IRFFN modules. The purpose of the local perception module [32] is to equip the model with the ability to extract local features while preserving its long-range capabilities. To avoid losing long-range information, a shortcut mechanism is incorporated. This particular element is visually highlighted in Figure 4 using yellow annotations. Next, we apply LMHSA computation to the feature transformation, represented in light green in Figure 4. To enhance the representation ability of tokens, we introduce the inverted residual feed-forward network module, or IRFFN, which can perform dimensional expansion and non-linear transformation on each token.

As described in [37], the original self-attention module utilizes linear mapping to derive the query matrix *Q*, key matrix *K*, and value matrix *V*. The dimensions of *Q*, *K* and *V* are given by *H × W × d_k_*, *H × W × d_k_* and *H × W × d_v_*, respectively, where *H × W* represents the number of image patches, and *d_k_* and *d_v_* are the dimensions of the tensor *K* and *V*. Subsequently, the self-attention module can be expressed as the following formula:

(3) $$Attn\left(Q,K,V\right)=Softmax\left(\frac{QK^{T}}{\sqrt{d_{k}}}\right)V$$

While the original self-attention algorithm can effectively handle various visual tasks, it is associated with high computational costs. As such, numerous researchers have dedicated efforts to addressing this issue. In our work, we adopt a similar approach to that in [37] which involves the use of *k × k* depth-wise convolutional operations with stride of *s* to reduce the dimensionality of the key and value matrices, thereby mitigating the computational burden. The computation associated with the depth-wise convolution can be expressed as follows:

(4) $$\{\begin{array}{c} Q=Linear\left(X\right)\;\\ K=Linear\left(DWConv\left(X\right)\right)\\ V=Linear\left(DWConv\left(X\right)\right) \end{array}$$

Equation (4) describes the lightweight self-attention mechanism used in our approach, where *X* represents the input feature, *DWConv*(·) denotes the depth-wise convolution operation, and *Linear*(·) is the linear operation. To further enhance performance, we incorporate a position bias term. Then the lightweight self-attention can be defined as:

(5) $$LightAttn\left(Q,K,V,B\right)=Softmax\left(\frac{QK^{T}}{\sqrt{d_{i}}}+B\right)V$$

**Figure 5:**
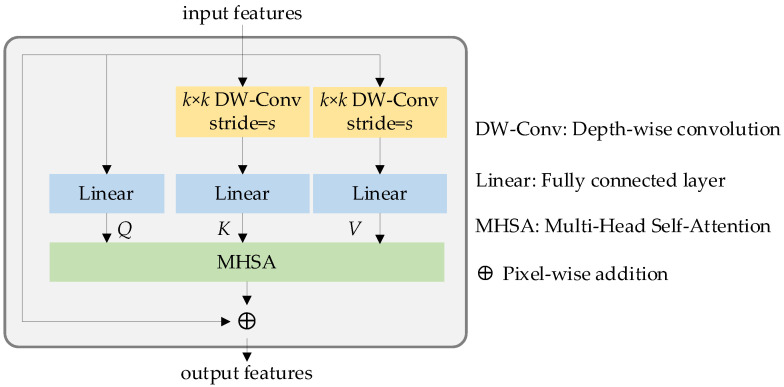
Lightweight multi-head self-attention block.

In the above formula, the dimensions of the query (*K*) and value (*V*) matrices are reduced to 1/*s*^2^ due to the application of stride *s* in the depth-wise convolution kernel. *d_i_* is the dimension of *Q*. The structure of the lightweight multi-head self-attention module is depicted in Figure 5.

**Figure 6:**
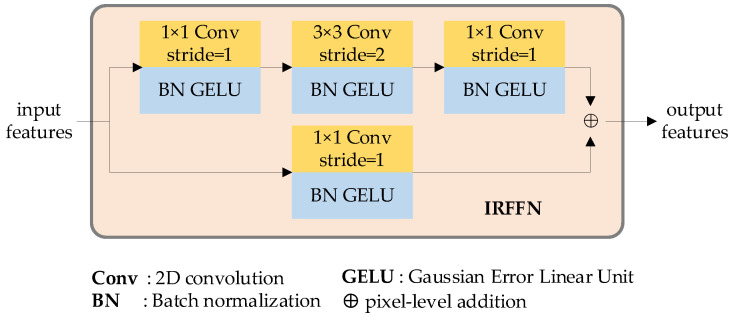
IRFFN block diagram.

In the robot grasp detection architecture, the IRFFN layer serves to expand and reduce feature dimensions, allowing for non-linear transformation. However, unlike the structure proposed in [32], we utilize an improved IRFFN layer to boost the expressiveness of the features. Figure 6 displays the structure of our proposed IRFFN layer which consists of two branches.

- Light weight multi-head cross-attention (LMHCA) module

The fusion algorithm we developed tackles the challenge of how to optimize the utilization of information from RGB and depth modalities, taking into account their respective importance. To further enhance the robustness of the fused features, we designed a cross-attention mechanism and a modal reweighting strategy. These techniques work in tandem to ensure that the most salient features from each modality are given the appropriate attention and weight in the final fusion result.

**Figure 7:**
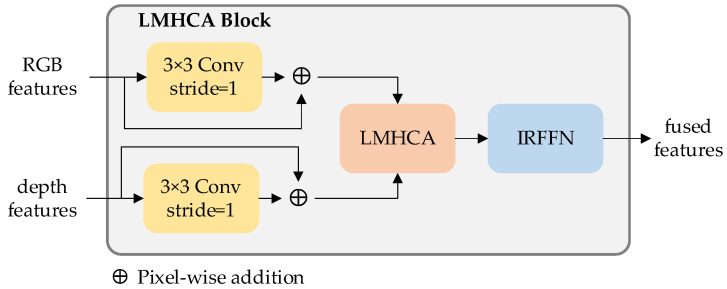
LMHCA block for fused feature extraction.

The proposed modal interaction strategy is illustrated in Figure 7. To address the issue of missing local associations and structural information during cross-attention computation, we incorporate local perception units into the design. Subsequently, multi-head cross-attention operations are employed to extract high-level semantic features from the RGB and depth features. Finally, the output features are fed into the IRFFN unit to enable dimensional expansion and non-linear transformation.

**Figure 8:**
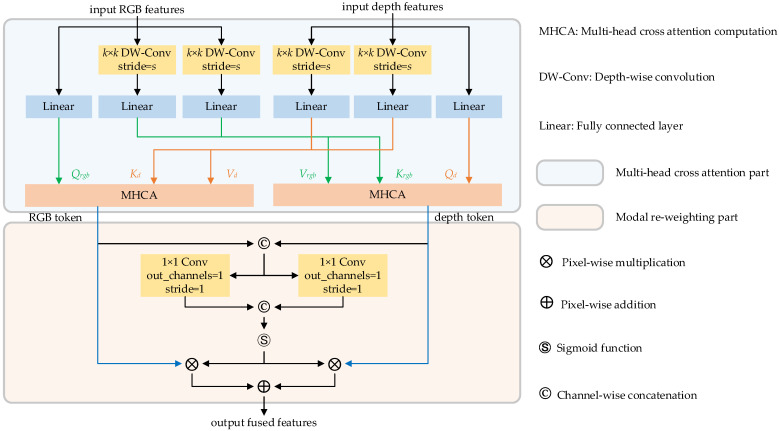
Multi-head cross-attention block diagram.

The token compounding method proposed by [38] has demonstrated outstanding performance in vision-and-language representation learning. Building upon this approach, we propose a lightweight multi-head cross-attention method to facilitate the fusion of RGB and depth features in robotic gripping applications. The lightweight multi-head cross-attention unit comprises two parts: a multi-head cross-attention component and a modality reweighting-based feature fusion component. Figure 8 presents a detailed schematic of the proposed procedure. Specifically, in the cross-attention module, we employ linear layer to acquire matrices *Q_rgb_*, *Q_d_* for RGB and depth streams, respectively. Depth-wise convolution and linear mapping are used to obtain the matrices *K_rgb_*, *V_rgb_*, *K_d_*, *V_d_* accordingly. Then, the lightweight cross-attention computation can be expressed as Equation (6).

(6) $$\{\begin{array}{c} LightCrossAtten\left(RGB2depth\right)=Softmax\left(\frac{Q_{rgb}K_{d}{}^{T}}{\sqrt{d_{i}}}+B\right)V_{d}\\ LightCrossAtten\left(depth2RGB\right)=Softmax\left(\frac{Q_{d}K_{rgb}{}^{T}}{\sqrt{d_{i}}}+B\right)V_{rgb} \end{array}$$

RGB and depth tokens are exported according to the upper operation. These two tokens are concatenated and fed into the modality reweighting process to create fused features. Different from the strategy of [39], we developed a novel modal reweighting method and assign appropriate adaptive weights to RGB and depth tokens, respectively, to obtain effective fused features. The detailed structure of the modality reweighting strategy can be seen in the bottom half of Figure 8. First, we concatenate the RGB and depth tokens, followed by using 1 × 1 convolution and SoftMax functions to learn adaptive weights for both token types. The fused features are obtained by reweighting the RGB and depth tokens and adding them at the pixel level.

### 4.3. Feature Aggregation Based on Channel Interaction Module (CIM)

In Figure 1, three feature maps cf7, df7 and ff7 can be obtained after the feature extraction procedures. These feature maps are subsequently aggregated for grasp prediction.

**Figure 9:**
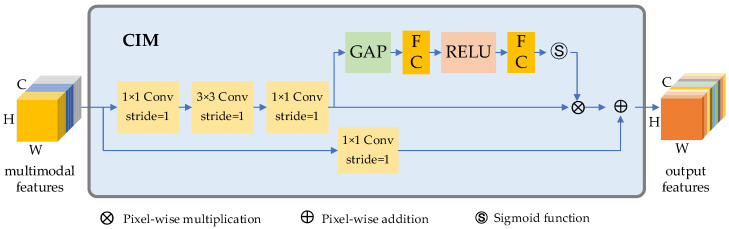
Channel interaction module.

Since each of these maps contains both valuable and non-valuable information, we apply the channel interaction method to reweight each channel of the connected features accordingly. To accomplish this, we use an SE-block [40] to improve the sensitivity of valuable channels and suppress useless ones. The structure of the CIM unit is shown in Figure 9.

To generate a feature map that is of the same size as the input image, we employ the same up-sampling techniques used in the feature decoding step in the final stages of the feature aggregation process.

### 4.4. Robot Grasp Prediction

As outlined in Section 3 and Section 4.1, the neural network we designed is expected to generate four heatmaps, namely $\tilde{Q}$, $\sin\left(2{\tilde{\emptyset }}_{i,j}\right)$, $\cos\left(2{\tilde{\emptyset }}_{i,j}\right)$ and $\tilde{W}$, to facilitate robot grasping. To accomplish this, four separate branches of 2-D convolutions are constructed.

### 4.5. Loss Function

We train the neural network by minimizing the discrepancy between the predicted grasps ($\tilde{G}$) and the ground truth grasps (*G*). To accomplish this, we utilize the smooth L1 loss function [41] in our work. This loss function is defined as follows:

(7) $$L\left(\tilde{G}-G\right)=\sum_{i}^{N}\sum_{k\in \left\{Q,\emptyset ,W\right\}}smooth_{L1}\left(\tilde{G_{i,j}^{k}}-G_{i,j}^{k}\right)$$

In Equation (7), *N* represents the number of pixels in the heatmap, and *smooth_L_*_1_(*x*) is defined as:

(8) $$smooth_{L1}\left(x\right)=\{\begin{array}{c} 0.5\times \frac{x^{2}}{\beta },\;if\left|x\right|<\beta \\ \left|x\right|-0.5\beta ,\;otherwise \end{array}$$

Here, the hyperparameter *β* controls the extent of smoothness and separates the positive axis range into *L*1 loss and *L*2 loss parts. In our work, we set the parameter *β* to 1.

## 5. Evaluation

### 5.1. Experimental Methodology

#### 5.1.1. Experiment Content

We designed four experiments to comprehensively evaluate the proposed method. The first two experiments are comparison studies that aim to verify the performance of different approaches on the Cornell and Jacquard datasets, respectively. The third experiment is an ablation study that examines the effects of cross-attention and channel interaction strategies. In this experiment, we evaluated the effectiveness of the modality adaptive reweighting algorithm in the fused feature extraction stage and the effects of the channel interaction algorithm in the feature aggregation stage. Additionally, we verified the effectiveness of the proposed algorithm through a fourth physical experiment.

#### 5.1.2. Datasets

We utilized two datasets, the Cornell dataset [1] and the Jacquard dataset [42], in our experiments. The Cornell dataset is relatively small, comprising 240 distinct objects with 885 samples, while the Jacquard dataset is of medium size, consisting of 11,619 unique objects and 54,485 different scenes. Both datasets provide RGB images and 3D dense cloud data for each sample. Prior to training the neural network, we converted the 3D point cloud data into depth images and adjusted their resolution to 224 × 224. We allocated 90% of each dataset for training and the remaining 10% for testing. Given the small size of the Cornell dataset, we augmented the dataset by performing augmentation operations such as cropping and rotation.

#### 5.1.3. Experiment Environment

The training and validation process was conducted on the Ubuntu 20.04 operating system, utilizing an Intel Core i9-12900KF CPU clocked up to 5.20 GHz, 64 GB DDR4 memory, and an NVIDIA GeForce GTX 3090-Ti graphics card. This computing server is manufactured by Kuankes Co., Shanghai, China.

**Figure 10:**
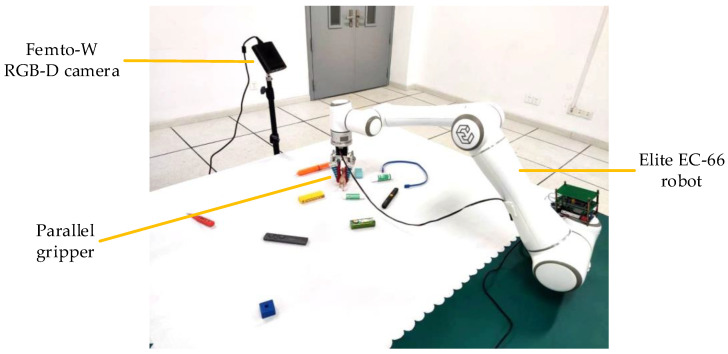
Physical experiment conditions. Experiment instruments include a Femto-W RGB-D camera, an EC-66 collaborative robot, a parallel gripper, and some objects to be grasped.

We set up a real-world robot grasp scenario, as depicted in Figure 10. For this experiment, we gathered 30 distinct objects on a desk. An Orbbec Femto-W RGBD camera was used as the image sensor, while a parallel gripper was installed at the end of the Elite EC-66 collaborative robot to act as the clamping mechanism. Prior to the experiment, a hand-eye calibration was performed to ensure proper operation of the system.

#### 5.1.4. Grasp Detection Metric

In all our experiments, we employ the grasp intersection over union (*IoU*) metric, which is defined in Equation (9). The use of this metric allows for a quantitative evaluation of the performance of our proposed method in terms of its ability to accurately predict grasps.

(9) $$IoU=\frac{G_{det}\cap^{\;}G_{GT}}{G_{det}\cup^{\;}G_{GT}}\times 100\%$$

The Intersection over Union (*IoU*) metric is utilized in all experiments, as defined in Equation (8). The numerator of the equation represents the area of overlap between the detected grasp rectangle and the ground truth, while the denominator represents their union. To be considered a valid detection, the detection results must exhibit the following properties:

- *IoU* between the detection result and the ground truth should be above 25%;
- The angle error between the detection result and the ground truth should be less than 30°.

In order to assess the overall detection accuracy, we conducted experiments on the validation dataset whereby we tabulated the number of grasping rectangles that fulfilled the specified criteria, as well as those that did not. This approach allowed for a thorough and precise evaluation of the detection performance.

#### 5.1.5. Experiment Configuration

Our proposed architecture has input dimensions of 224 × 224 × 3 and 224 × 224 × 1 for RGB and depth images, respectively. The detailed size of each feature map is listed in Table 1.

We utilized self-attention and cross-attention based feature encoders with 1, 2, 4, and 8 heads, and the corresponding number of block layers are 2, 2, 10, and 2, respectively.

To train the proposed neural network, AdamW stochastic gradient descent was used with the batch size of 16. We implemented a warm-up and multi-step learning rate scheduler. The maximum learning rate was configured to 1 × 10^−4^, with the learning rate being adjusted every 10 epochs. The multiplicative factor for the learning rate decay was set to 0.5, ensuring optimal performance and stability during training. During the training process using the Jacquard dataset, the neural network was initialized with random parameters and subsequently trained over 15 epochs. Each epoch consisted of 3065 batches. In the subsequent training procedure using the Cornell dataset, the neural network’s hyperparameters were initialized with the parameters previously trained on the Jacquard dataset.

### 5.2. Experiement Results

#### 5.2.1. Cornell Dataset Experiment Results

To compare the grasp detection performance of recent methods with our proposed algorithm on the Cornell dataset, we conducted an experiment that evaluated image-wise and object-wise grasp detection separately. Our algorithm achieved state-of-the-art accuracy of 99.4% and 97.8% in image-wise and object-wise grasp detection, respectively, as shown in Table 2. However, the average time expenditure is 17.7 ms, which is higher compared to algorithms in [10,11,12] due to the complexity of our algorithm.

**Figure 11:**
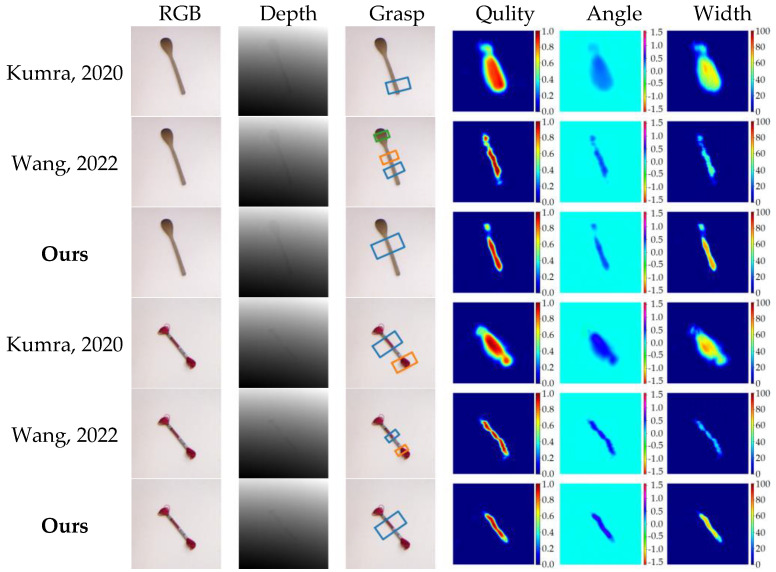
Experiment results of the algorithms proposed by Kumra et al. [8] in 2020, Wang et al. [6] in 2022 and our method on Cornell dataset. The 1st and 2nd columns are RGB image and depth images. The 3rd column shows grasp detection results. The last three columns illustrate the quality, angle, and width heatmaps.

Figure 11 depicts some typical examples of heatmap regression results for quality, angle, and width, as well as grasp detection results. As shown in the figure, the quality heatmaps demonstrate the robustness of our proposed method, which contributes to the superior performance of our grasp detection results.

#### 5.2.2. Jacquard Dataset Experiment Results

We also conducted a comparative analysis of our grasp detection algorithm with that of several other methods [5,6,8,9,10,11,12] using the Jacquard dataset. Table 3 presents the statistical results of our experiment with the Jacquard dataset. As is evident from the table, our algorithm achieved the highest image-wise and object-wise detection accuracy of 96.7% and 94.6%, respectively, on the Jacquard dataset. Figure 11 shows several detection cases. Our algorithm offers superior quality heatmap prediction results.

**Figure 12:**
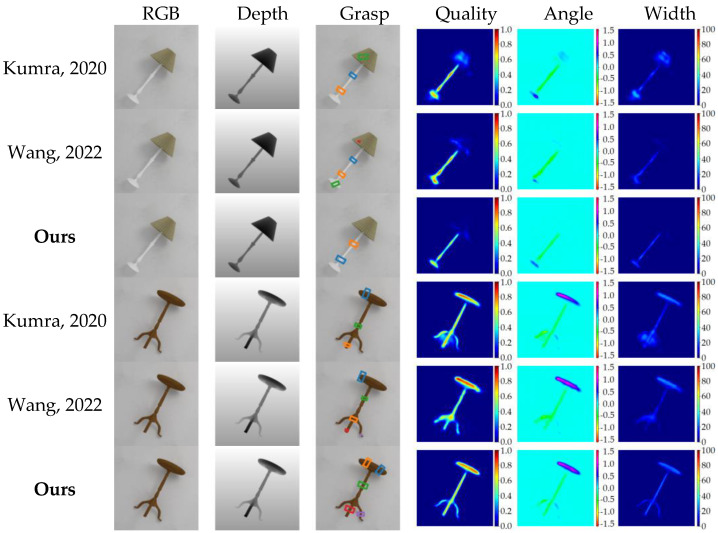
Experiment results of algorithms proposed by Kumra et al. [8] in 2020, Wang et al. [6] in 2022 and our method on Jacquard dataset.

The detection results presented in Table 3 and Figure 12 provide evidence that our proposed method, which leverages cross-attention and channel interaction for RGB-D feature fusion, can effectively utilize the information shared between the two modalities.

#### 5.2.3. Ablation Experiment

**Figure 13:**
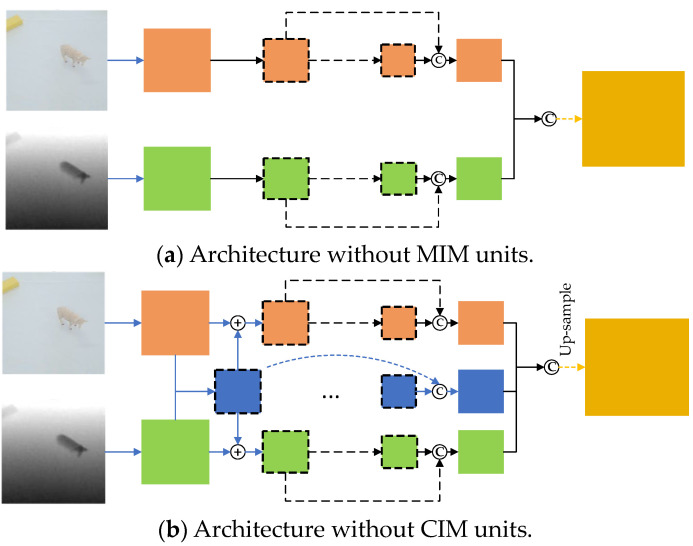
Architectures in ablation experiments: (**a**) has no MIM blocks; and (**b**) has no CIM blocks.

Since the cross-attention module is only involved in the fused feature encoding stage, we simplified our pipeline (shown in Figure 1) to produce the architecture depicted in Figure 13.

In the ablation experiment, we evaluated the object-wise grasp detection accuracy on both the Cornell and Jacquard datasets. The corresponding statistical results are presented in Table 4.

To validate the effectiveness of the different modules in the proposed approach, we conducted several leave-one-out experiments on the Cornell and Jacquard datasets. Initially, we removed the MIM and CIM modules from the proposed architecture. The generated approaches are served as baseline approaches, shown in Figure 13a,b.

The results of the ablation experiment demonstrate that the bilateral modality interaction method based on cross-attention significantly enhances the accuracy of grasp detection. Additionally, the feature aggregation method based on channel interaction strategy has a fine-tuning effect on detection accuracy.

#### 5.2.4. Physical Experiment

**Figure 14:**
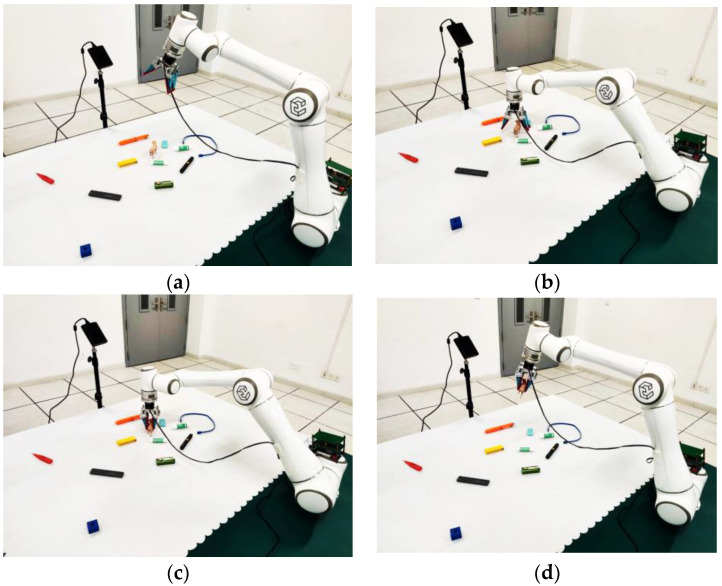
Four grasp stages in the physical experiment: (**a**) shows the initial position and posture of EC-66 robot. In this stage, grasp detection is performed. After detection, the parallel gripper moves to the effective grasping position, which can be seen in (**b**). The gripper then grasps the target, which is shown in (**c**). The robot completes the target grasping task in (**d**) finally.

The physical experiment was conducted on our in-house robotic platform, which comprises an Elite EC-66 robot with public open ROS interfaces, a parallel gripper, an Orbbec Femo-W RGB-D camera, and a computer server running Ubuntu. The experiment involved 30 different unknown objects in the scene, with the camera positioned relative to the desktop similar to that in the Cornell dataset. RGB-D image data was captured by the camera, and the server detected the position and pose of potential grasps. Following a coordinate transformation, the robot executed the grasping operation on the target object. The detailed grasp process is depicted in Figure 14.

**Figure 15:**
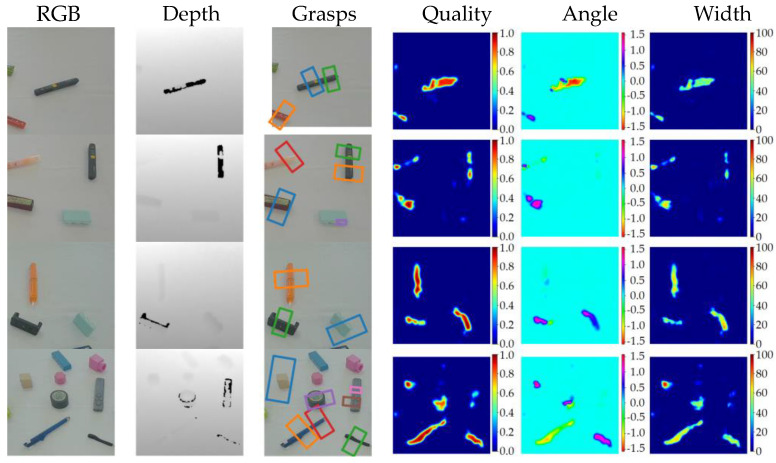

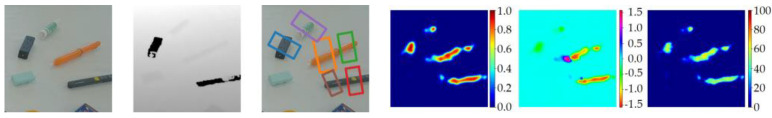
Detection results of the physical experiment.

During the experiment, we made a total of 200 attempts to grasp the target objects and successfully grasped the objects in 189 of those attempts, resulting in an average success rate of 94.5%. Figure 15 illustrates several examples of our grasp detection results.

## 6. Conclusions

This paper addressed the 2-DoF robot grasp detection problem by analyzing data fusion issues that affect grasp detection results. Our analysis showed that fully utilizing useful information from each modality and eliminating useless information is essential for achieving high accuracy in grasp detection. In response, we proposed a cross-modality fusion method for 2-DoF robot grasp detection that used a convolutional neural network and transformer structure. This method incorporated modal interaction and channel interaction strategies to adaptively retain essential information and reduce the impact of invalid information. To validate our approach, we conducted a series of comparison experiments with other methods and an ablation experiment. Our experimental results demonstrated that the proposed cross-modality fusion method achieves high accuracy for both image-wise and object-wise grasp detection and is effective in practical robot grasp detection scenarios. However, this method is somewhat time-consuming, which is a limitation that we will address in future work.

Our proposed algorithm has demonstrated applicability to the task of detecting targets for robotic grasping in vision-based scenarios. In addition, we anticipate that our work will yield valuable insights into RGB-D fusion, with potential applications in image semantic segmentation, target detection, and pose estimation.

## Figures and Tables

**Table 1:** Size of each feature map.

Feature Map	Size (H × W × C)	Feature Map	Size (H × W × C)
cf0, df0	112 × 112 × 16	cf5, df5, ff5	14 × 14 × 184
cf1, df1, ff1	56 × 56 × 46	cf6, df6, ff6	28 × 28 × 92
cf2, df2, ff2	28 × 28 × 92	cf7, df7, ff7	56 × 56 × 46
cf3, df3, ff3	14 × 14 × 184	f8	112 × 112 × 46
cf4, df4, ff4	7 × 7 × 368	f9	224 × 224 × 32

**Table 2:** Grasp detection results of different algorithms on Cornell dataset.

Method	Input	Accuracy (%)		Time (ms)
		Image-Wise	Object-Wise	
Lenz [1]	RGB-D	73.9	75.6	1350
Redmon [27]	RGB-D	88	87.1	76
Morrision [5]	D	73	69	19
Song [10]	RGB-D	92.5	90.3	17.2
Kumra [8]	RGB-D	97.7	96.6	20
Wang [6]	RGB-D	97.99	96.7	41.6
Yu [9]	RGB-D	98.2	97.1	25
Tian [11]	RGB-D	98.9	-	15
Tian [12]	RGB-D	99.3	91.1	12
Ours	RGB-D	99.4	97.8	17.7

**Table 3:** Grasp detection results of different algorithms on Jacquard dataset.

Method	Input	Accuracy (%)	
		Image-Wise	Object-Wise
Morrison [5]	D	84	-
Song [10]	RGB-D	93.2	-
Kumra [8]	RGB-D	92.6	87.7
Wang [6]	RGB-D	94.6	-
Yu [9]	RGB-D	95.7	-
Tian [11]	RGB-D	94	-
Tian [12]	RGB-D	94.6	92.8
Ours	RGB-D	96.7	94.6

**Table 4:** Object-wise grasp detection results of ablation experiment on Cornell and Jacquard datasets.

Methods	Accuracy of Cornell Dataset (%)	Accuracy of Jacquard Dataset (%)
Without MIM	89.7	84.6
Without CIM	96.4	92.6
With MIM and CIM	97.8	94.6

## Data Availability

Cornell, Jacquard and multi-object multi-grasp RGB-D dataset experiment results are available on https://github.com/QZhSSLab/CrossModalityFusion4RobotGrasp, since 9 February 2022. The code presented in this study is available on request from the corresponding author.

## References

[1] Lenz I., Lee H., Saxena A. (2015). Deep Learning for Detecting Robotic Grasps. Int. J. Robot. Res..

[2] Zhang Q., Qu D., Xu F., Zou F. (2017). Robust Robot Grasp Detection in Multimodal Fusion. MATEC Web Conf..

[3] Cao H., Chen G., Li Z., Feng Q., Lin J., Knoll A. (2022). Efficient Grasp Detection Network with Gaussian-Based Grasp Representation for Robotic Manipulation. IEEE ASME Trans. Mechatron..

[4] Morrison D., Corke P., Leitner J. (2018). Closing the Loop for Robotic Grasping: A Real-Time, Generative Grasp Synthesis Approach. Robotics.

[5] Morrison D., Corke P., Leitner J. (2019). Learning Robust, Real-Time, Reactive Robotic Grasping. Int. J. Robot. Res..

[6] Wang S., Zhou Z., Kan Z. (2022). When Transformer Meets Robotic Grasping: Exploits Context for Efficient Grasp Detection. IEEE Robot. Autom. Lett..

[7] Chu F.-J., Xu R., Vela P.A. (2018). Real-World Multiobject, Multigrasp Detection. IEEE Robot. Autom. Lett..

[8] Kumra S., Joshi S., Sahin F. Antipodal Robotic Grasping Using Generative Residual Convolutional Neural Network. Proceedings of the 2020 IEEE/RSJ International Conference on Intelligent Robots and Systems (IROS).

[9] Yu S., Zhai D.-H., Xia Y., Wu H., Liao J. (2022). SE-ResUNet: A Novel Robotic Grasp Detection Method. IEEE Robot. Autom. Lett..

[10] Song Y., Wen J., Liu D., Yu C. (2022). Deep Robotic Grasping Prediction with Hierarchical RGB-D Fusion. Int. J. Control Autom. Syst..

[11] Tian H., Song K., Li S., Ma S., Yan Y. (2022). Lightweight Pixel-Wise Generative Robot Grasping Detection Based on RGB-D Dense Fusion. IEEE Trans. Instrum. Meas..

[12] Tian H., Song K., Li S., Ma S., Yan Y. (2023). Rotation Adaptive Grasping Estimation Network Oriented to Unknown Objects Based on Novel RGB-D Fusion Strategy. Eng. Appl. Artif. Intell..

[13] Saxena A., Driemeyer J., Kearns J., Ng A. (2006). Robotic Grasping of Novel Objects. Advances in Neural Information Processing Systems.

[14] Le Q.V., Kamm D., Kara A.F., Ng A.Y. Learning to Grasp Objects with Multiple Contact Points. Proceedings of the 2010 IEEE International Conference on Robotics and Automation.

[15] Liang H., Ma X., Li S., Gorner M., Tang S., Fang B., Sun F., Zhang J. PointNetGPD: Detecting Grasp Configurations from Point Sets. Proceedings of the 2019 International Conference on Robotics and Automation (ICRA).

[16] Gou M., Fang H.-S., Zhu Z., Xu S., Wang C., Lu C. RGB Matters: Learning 7-DoF Grasp Poses on Monocular RGBD Images. Proceedings of the 2021 IEEE International Conference on Robotics and Automation (ICRA).

[17] Sundermeyer M., Mousavian A., Triebel R., Fox D. Contact-GraspNet: Efficient 6-DoF Grasp Generation in Cluttered Scenes. Proceedings of the 2021 IEEE International Conference on Robotics and Automation (ICRA).

[18] Jiang Y., Moseson S., Saxena A. Efficient Grasping from RGBD Images: Learning Using a New Rectangle Representation. Proceedings of the 2011 IEEE International Conference on Robotics and Automation.

[19] Shi C., Miao C., Zhong X., Zhong X., Hu H., Liu Q. (2022). Pixel-Reasoning-Based Robotics Fine Grasping for Novel Objects with Deep EDINet Structure. Sensors.

[20] Kumra S., Joshi S., Sahin F. (2022). GR-ConvNet v2: A Real-Time Multi-Grasp Detection Network for Robotic Grasping. Sensors.

[21] Li Z., Liu F., Yang W., Peng S., Zhou J. (2021). A Survey of Convolutional Neural Networks: Analysis, Applications, and Prospects. IEEE Trans. Neural Netw. Learn. Syst..

[22] Caldera S., Rassau A., Chai D. (2018). Review of Deep Learning Methods in Robotic Grasp Detection. Multimodal Technol. Interact..

[23] Kumra S., Kanan C. Robotic Grasp Detection Using Deep Convolutional Neural Networks. Proceedings of the 2017 IEEE/RSJ international conference on intelligent robots and systems (IROS).

[24] Wei J., Liu H., Yan G., Sun F. (2017). Robotic Grasping Recognition Using Multi-Modal Deep Extreme Learning Machine. Multidimens. Syst. Signal Process..

[25] Trottier L., Giguère P., Chaib-draa B. (1606). Dictionary Learning for Robotic Grasp Recognition and Detection. arXiv.

[26] Wang Z., Li Z., Wang B., Liu H. (2016). Robot Grasp Detection Using Multimodal Deep Convolutional Neural Networks. Adv. Mech. Eng..

[27] Redmon J., Angelova A. Real-Time Grasp Detection Using Convolutional Neural Networks. Proceedings of the 2015 IEEE International Conference on Robotics and Automation (ICRA).

[28] Ainetter S., Fraundorfer F. End-to-End Trainable Deep Neural Network for Robotic Grasp Detection and Semantic Segmentation from Rgb. Proceedings of the 2021 IEEE International Conference on Robotics and Automation (ICRA).

[29] Redmon J., Divvala S., Girshick R., Farhadi A. You Only Look Once: Unified, Real-Time Object Detection. Proceedings of the IEEE Conference on Computer Vision and Pattern Recognition.

[30] Ronneberger O., Fischer P., Brox T., Navab N., Hornegger J., Wells W.M., Frangi A.F. (2015). U-Net: Convolutional Networks for Biomedical Image Segmentation. Proceedings of the Medical Image Computing and Computer-Assisted Intervention—MICCAI 2015.

[31] Song K., Wang J., Bao Y., Huang L., Yan Y. (2022). A Novel Visible-Depth-Thermal Image Dataset of Salient Object Detection for Robotic Visual Perception. IEEEASME Trans. Mechatron..

[32] Guo J., Han K., Wu H., Tang Y., Chen X., Wang Y., Xu C. CMT: Convolutional Neural Networks Meet Vision Transformers. Proceedings of the 2022 IEEE/CVF Conference on Computer Vision and Pattern Recognition (CVPR).

[33] Xiao T., Singh M., Mintun E., Darrell T., Dollár P., Girshick R. Early Convolutions Help Transformers See Better. Proceedings of the Advances in Neural Information Processing Systems.

[34] He T., Zhang Z., Zhang H., Zhang Z., Xie J., Li M. Bag of Tricks for Image Classification with Convolutional Neural Networks. Proceedings of the 2019 IEEE/CVF Conference on Computer Vision and Pattern Recognition (CVPR).

[35] Hendrycks D., Gimpel K. (1606). Gaussian Error Linear Units (Gelus). arXiv.

[36] Ioffe S., Szegedy C. Batch Normalization: Accelerating Deep Network Training by Reducing Internal Covariate Shift. Proceedings of the International Conference on Machine Learning.

[37] Dosovitskiy A., Beyer L., Kolesnikov A., Weissenborn D., Zhai X., Unterthiner T., Dehghani M., Minderer M., Heigold G., Gelly S. (2010). An Image Is Worth 16x16 Words: Transformers for Image Recognition at Scale. arXiv.

[38] Aladago M.M., Piergiovanni A.J. (2022). Compound Tokens: Channel Fusion for Vision-Language Representation Learning. arXiv.

[39] Zhang Y., Choi S., Hong S. Spatio-Channel Attention Blocks for Cross-Modal Crowd Counting. Proceedings of the Asian Conference on Computer Vision.

[40] Hu J., Shen L., Sun G. Squeeze-and-Excitation Networks. Proceedings of the IEEE Conference on Computer Vision and Pattern Recognition.

[41] Ren S., He K., Girshick R., Sun J. Faster R-CNN: Towards Real-Time Object Detection with Region Proposal Networks. Proceedings of the Advances in Neural Information Processing Systems.

[42] Depierre A., Dellandréa E., Chen L. Jacquard: A Large Scale Dataset for Robotic Grasp Detection. Proceedings of the 2018 IEEE/RSJ International Conference on Intelligent Robots and Systems (IROS).

